# Supplementary material for: Environment, taxonomy, and socioeconomics predict non-imperilment in freshwater fishes
Source: Nat Commun. 2026 Feb 16;17:1661. doi: 10.1038/s41467-025-68154-w (PMC12909873; doi:10.1038/s41467-025-68154-w)
Supplement: Supplementary file 1 — Supplementary Information [file 41467_2025_68154_MOESM1_ESM.pdf]

Supplementary Information

to accompany

Environment, taxonomy, and socioeconomics predict non-imperilment in freshwater fishes

Christina A. Murphy[1]+*, J. Andres Olivos[2]+, Ivan Arismendi[3], Emili García-Berthou[4], Sherri L. Johnson[5], Jason Dunham[6]

[1] U.S. Geological Survey, Maine Cooperative Fish and Wildlife Research Unit, Orono, Maine, USA
[2] Department of Forest Engineering, Resources, and Management, Oregon State University, Corvallis, Oregon, USA 3
[3] Department of Fisheries, Wildlife, and Conservation Sciences, Oregon State University, Corvallis, Oregon, USA
[4] GRECO, Institute of Aquatic Ecology, University of Girona, Girona, Catalonia, Spain
[5] Pacific Northwest Research Station, USDA Forest Service, Corvallis, Oregon, USA
[6] U.S. Geological Survey, Forest and Rangeland Ecosystem Science Center, Corvallis, OR, USA
+these authors contributed equally
*christina.murphy@maine.edu

Any use of trade, firm, or product names is for descriptive purposes only and does not imply endorsement by the U.S. Government.

Table Captions

Table S1. Metadata including Categories
Metadata including categorization (Broad Category and Sub-category) of predictor variables (Field) preliminary compiled to inform a random forest model of IUCN species status for freshwater fishes. Note that not all variables were finally used for the random forest models. Incl = Y indicates predictors included in the final random forest model; N indicates predictors omitted from the random forest model.

Table S2. Summary Ordinal Forest
Summary of the ordinal forest model implemented using the 'cforest' algorithm in the party package (SR1) for 5 categories (Least Concern, LC; Near Threatened, NT; Vulnerable, VU; Endangered, EN; and Critically Endangered, CR). Confusion matrix (top) and predictive performance metrics by class (middle) for the final model as well as Performance metrics and variability in tuning (lower) with the final model highlighted in green. mtry = the number of variables (features) randomly sampled at each split, macro = macro-averaged, MAE = Mean Absolute Error, MSE = Mean Standard Error.

Table S3. Summary Random Forest
Summary of the binary categorical random forest model implemented using the randomForest package (SR2) to predict imperilment (Vulnerable, Endangered, and Critically Endangered) and non-imperilment (Least Concern and Near Threatened). Confusion matrix (top) and performance metrics and variability in tuning (bottom), with the final model highlighted in green. mtry = the number of variables (features) randomly sampled at each split, macro = macro-averaged, MAE = Mean Absolute Error, MSE = Mean Standard Error.

Table S4. Variable Importance
Variable importance metrics (VIMs) for ordinal forests based on various metrics (Error Rate, ER; Rank Probability Score, RPS; Mean Absolute error, MAE; Mean Squared Error, MSE) (SR3) and random forests (loss in area under the precision-recall curve, AUC-loss; class-specific Mean Decrease in Accuracy, MDA; Mean Decrease in Balanced Accuracy, MDBA; and Gini coefficient). Variables were ranked by predictive importance in the selected random forest model based on the AUC-loss metric.

Table S5. Partial Dependence on Binary Classification Model
Partial dependence plots of the final random forest model constructed using the 'pdp' package in R (SR4). Plots depict the average relationship between selected variables and the assignment probability to the imperilment class. Rows are ordered by importance based on precision-recall AUC (loss).

Supplemental References

SR1. Hothorn, T., Hornik, K., Strobl, C., Zeileis, A. & Hothorn, M. T. Package 'party'. Package Ref. Man. Party Version 09-998 16, 37 (2015).

SR2. Cutler, F. original by L. B. and A. & Wiener, R. port by A. L. and M. randomForest: Breiman and Cutler's Random Forests for Classification and Regression. (2015).

SR3. Janitza, S., Tutz, G. & Boulesteix, A.-L. Random forest for ordinal responses: Prediction and variable selection. Comput. Stat. Data Anal. 96, 57–73 (2016).

SR4. Greenwell, B. M. pdp: An R package for constructing partial dependence plots. R J 9, 421 (2017).

Code Availability

Scripts and tables can be accessed at https://github.com/AndresOlivos/freshwater_fish_imperilment_classification/

S1. Metadata including Categories

Broad Category	Sub-category	Includ.	Field	Meaning	Unit	Source	Reference
Environmental	Habitat	N	Perimeter	Species range perimeter	km	International Union for Conservation of Nature (IUCN) RedList	International Union for Conservation of Nature (IUCN). The IUCN Red List of Threatened Species. Version 2024-2. (2024).
		N	Perimeter_p	Species range perimeter / range area	km^2 / area		
		N	North_Limit	Species range northern limit	Decimal Degrees		
		Y	South_Limit	Species range southern limit	Decimal Degrees		
		Y	East_Limit	Species range eastern limit	Decimal Degrees		
Socioeconomic	Impoundments	N	Dams	Total existing dams within range	Count	Global Dam Watch (GDW)	Lehner, B. *et al.* The Global Dam Watch database of river barrier and reservoir information for large-scale applications. *Sci. Data* **11**, 1069 (2024).
		Y	Dams_p	Total existing dams / range area	Count		
		N	CAB_Dams_p	Total catchment areas blocked	km^2		
		Y	CAB_Dams_p	Total catchment areas blocked / range area	Proportion		
		N	DB_Dams	Total discharge blocked	m^3/sec		
		Y	DB_Dams_p	Total discharge blocked / range area	m^3/sec / area		
		N	RA_Dams	Total reservoir area in range	km^2		
		Y	RA_Dams_p	Total reservoir area in range / range area	Proportion		
	Economy	Y	GDPslope	Avg. annual change in GDP	Million USD	World Bank	The World Bank. World Development Indicators. World Bank Open Data Catalog. Washington, D.C., United States. (2020).
		Y	GDPpercent	Avg. annual percent change in GDP	Percent		
		N	PCslope	Avg. annual change in GDP per capita	Million USD		
		N	PCpercent	Avg. annual percent change in GDP per capita	Percent		
		Y	EarlyPC	Early GDP per capita (1960s)	Million USD		
	Development	Y	PDslope	Avg. annual change in population density 2000-2020	Population/km^2	WorldPop	Lloyd, C. T. *et al.* Global spatio-temporally harmonised datasets for producing high-resolution gridded population distribution datasets. *Big Earth Data* **3**, 108–139 (2019).
		Y	PDpercent	Avg. annual percent change in population density	Percent Change		
		N	LatestPD	Population density 2020	Population/km^2		
		N	EarlyPD	Population density 2000	Population/km^2		
Environmental	Climate	N	bio_1	Historic Annual Mean Temperature	°C	WorldClim v2.1	Fick, S. E. & Hijmans, R. J. WorldClim 2: new 1-km spatial resolution climate surfaces for global land areas. *Int. J. Climatol.* **37**, 4302–4315 (2017).
		N	bio_2	Historic Mean Diurnal Temperature Range	°C		
		N	bio_3	Historic Isothermality	°C (Ratio)		
		Y	bio_4	Historic Temperature Seasonality	°C (SD)		
		N	bio_5	Historic Max Temperature of Warmest Month	°C		
		N	bio_ 6	Historic Min Temperature of Coldest Month	°C		
		N	bio_7	Historic Temperature Annual Range	°C		
		Y	bio_8	Historic Mean Temperature of Wettest Quarter	°C		
		N	bio_9	Historic Mean Temperature of Driest Quarter	°C		
		N	bio_10	Historic Mean Temperature of Warmest Quarter	°C		
		N	bio_11	Historic Mean Temperature of Coldest Quarter	°C		
		N	bio_12	Historic Annual Precipitation	mm		
		N	bio_13	Historic Precipitation of Wettest Month	mm		
		N	bio_14	Historic Precipitation of Driest Month	mm		
		N	bio_15	Historic Precipitation Seasonality	mm		
		Y	bio_16	Historic Precipitation of Wettest Quarter	mm		
		Y	bio_17	Historic Precipitation of Driest Quarter	mm		
		Y	bio_18	Historic Precipitation of Warmest Quarter	mm		
		Y	bio_19	Historic Precipitation of Coldest Quarter	mm		

S1. Metadata including Categories (continued)

Broad Category	Sub-category	Includ.	Field	Meaning	Unit	Source	Reference
Species	Physiology	N	pH_Range	maximum – minimum pH	standard units	Fish Base	Froese, R. & Pauly, D. FishBase. World Wide Web electronic publication, version (04/2025). (2025).
		N	pHMin	Minimum pH	standard units		
		N	pHMax	Maximum pH	standard units		
		N	dH_Range	dH_range	water hardness		
		N	dHMin	dHMin	water hardness		
		N	dHMax	dHMax	water hardness		
	Life history	Y	BodyShapeI	Body form	categorical	IUCN RedList	
	Physiology	Y	Brack	Use of brackish water	presence		
Species		Y	Saltwater	Use of saltwater	presence	Fish Base	
		Y	DemersPelag	Demersal/Pelagic	categorical		
	Life history	N	AnaCat	Diadromous migration	categorical		
	Physiology	N	DepthRangeShallow	Shallow end of depth range	m		
		N	DepthRangeDeep	Deep end of depth range	m		
	Life history	N	LongevityWild	Lifespan	years		
Socioeconomic	Value	N	Importance	commercial importance	categorical		
Response	Endangerment	N	Vulnerability	Vulnerability	categorical		
Species	Life history	Y	LengthCategory	Small, medium, large	categorical		
		N	Weight	Mass	g		
Socioeconomic	Value	Y	UsedforAquaculture	Aquaculture	categorical		
		Y	UsedasBait	Used as bait	categorical		
		Y	Aquarium	Aquarium trade	categorical		
		Y	GameFish	Game fish	presence		
Species	Life history	Y	Dangerous	Dangerous	categorical		
		Y	Electrogenic	Electric	categorical		
		N	FeedingType	Feeding method	categorical		
		N	TrophicLevel	Trophic level	trophic level		
	Physiology	N	UseTemp	Temperature preference	°C		
		N	TRange	Thermal range	°C		
	Life history	N	AdultMode	Locomotion	categorical		
	Taxonomic	Y	Order	Taxonomic order	categorical		
	Physiology	N	Organic.material	Habitat substrate organic material	presence		
		N	Coarse.sand.or.finer	Habitat substrate sand and fines	presence		
		N	Rocky.bottom	Habitat substrate rocky	presence		
Response	Endangerment	N	population_trend	Population trend	categorical	IUCN RedList	
Species	Physiology	N	IUCN.temporary	Habitat temporary	presence		
		N	IUCN.permanent	Habitat permanent	presence		
		N	IUCN.nat.lentic	Habitat natural lentic	presence		
		N	IUCN.nat.lotic	Habitat natural lotic	presence		
		N	IUCN.artificial	Habitat artificial	presence		
		N	IUCN.other	Other habitats	presence		
Socioeconomic	Management	N	M_Habitat	Management recommendation habitat	presence		
		N	M_Species	Management recommendation species	presence		
		N	M_SocioeconomicPolicy	Management recommendation Socioeconomic policy	presence		
	Knowledge	Y	na_count	Knowledge gaps	count	FishBase + IUCN	

S1. Metadata including Categories (continued)

Broad Category	Sub-category	Includ.	Field	Meaning	Unit	Source	Reference
Environmental	Climate	Y	Mean_Elevation	Average elevation of occurrence locations	masl	Global Biodiversity Information Facility (GBIF)	GBIF. https://www.gbif.org/
		N	Min_Elevation	Minimum elevation of occurrence locations	masl		
		N	Max_Elevation	Maximum elevation of occurrence locations	m asl		
	Habitat	N	Class_count	Count of different classes of geomorphic sub-classification	count	Global River Classification (GloRiC)	Ouellet Dallaire, C., Lehner, B., Sayre, R. & Thieme, M. A multidisciplinary framework to derive global river reach classifications at high spatial resolution. *Environ. Res. Lett.* **14**, 024003 (2019).
		Y	Class_count_p	Count of different classes of geomorphic sub-classification / area species range	count / area		
		Y	Class_geom	Mode of different classes of geomorphic sub-classification	categorical		
		Y	Class_hydr	Mode of classes of hydrologic sub-classification	categorical		
		Y	Class_phys	Mode of classes of physio-climatic sub-classification	categorical		
	Hydrology	N	Lake_wet	Count of lake and wetland reaches	count		
		N	Lake_wet_p	Count of lake and wetland reaches / area of species range	count		
		Y	Log_Q_avg	$\log_{10}$ of long-term average discharge	m^3/sec		
		Y	Log_Q_var	Flow regime variability as $\log_{10}$ max. monthly avg. / long-term avg. discharge	index		
		Y	Reach_type	Mode of reach type (4 classes)	categorical		
		Y	Stream_pow	Total stream power	kW/m^2		
	Climate	N	Temp_min	Mean long-term average of the minimum air temperature of the coldest month	°C	GloRiC	
		Y	CMI_indx	Mean reach climate moisture index	index		
	Habitat	N	LC_Bare	Cover type percentage	percent	Copernicus - PROBA-V	Copernicus - PROBA-V. https://dataspace.copernicus.eu/proba-v
Socioeconomic	Development	Y	LC_Crops	Cover type percentage	percent		
Environmental	Habitat	Y	LC_Grass	Cover type percentage	percent		
		Y	LC_Shrub	Cover type percentage	percent		
	Hydrology	Y	LC_Snow	Cover type percentage	percent		
	Habitat	Y	LC_Trees	Cover type percentage	percent		
		Y	LC_PermanentWater	Cover type percentage	percent		
		Y	LC_SeasonalWater	Cover type percentage	percent		
Socioeconomic	Footprint	N	hfp_1993	Human-footprint index in 1993	index	Global Terrestrial Human Footprint	Venter, O. *et al.* Global terrestrial Human Footprint maps for 1993 and 2009. *Sci. Data* **3**, 1–10 (2016).
		Y	hfp_2009	Human-footprint index in 2009	index		
Environmental	Habitat	Y	EnvTemp	Tropical, subtropical, temperate	categorical	Fish Base	
		N	Symp_count	count of overlapping ranges of other species	count	IUCN RedList	
		N	Symp_count_p	count of overlapping ranges of other IUCN species / size of range of species	count / area		
		N	Intro_count_p	count of introduced taxa in range / area of species range	count / area		
		N	Intro_count	count of introduced taxa in range	count		
		Y	Extir_count	count of extirpated species in range	count		
Socioeconomic	Footprint	N	POP_SERVED	human population wastewater service	population	HydroWASTE	Ehalt Macedo, H. *et al.* Distribution and characteristics of wastewater treatment plants within the global river network. *Earth Syst. Sci. Data* **14**, 559–577 (2022).
		Y	POP_SERVED_p	human population wastewater service/area of species range	population		
		N	WASTE_DIS	sewage discharge	m^3/sec		
		N	WASTE_DIS_p	sewage discharge/area of species range	m^3/sec / area		
	Conservation	Y	Protected_Percent	Protected area/area of species range	%	World Database of Protected Areas (WDPA)	UNEP-WCMC, I. Protected planet: The world database on protected areas. *UNEP-WCMC Camb. UK* (2025).
		N	Ramsar_count	Number of Ramsar sites within species range	count		
		Y	Ramsar_count_p	Number of Ramsar sites/area of species range	count / area		

S2. Summary of Ordinal Forest

Confusion matrix

		Reference				
		**LC**	**NT**	**VU**	**EN**	**CR**
**Predictions**	**LC**	1443	113	130	97	35
	**NT**	0	1	0	0	0
	**VU**	10	2	29	10	4
	**EN**	12	21	47	97	45
	**CR**	2	1	4	7	22

Predictive performance metrics by class

	Sensitivity	Specificity	Pos Pred Value	Neg Pred Value	Precision	Recall	F1	Prevalence	Detection Rate	Detection Prevalence	Balanced Accuracy
**LC**	0.98	0.44	0.79	0.92	0.79	0.98	0.88	0.69	0.68	0.85	0.71
**NT**	0.01	1.00	1.00	0.94	1.00	0.01	0.01	0.06	0.00	0.00	0.50
**VU**	0.14	0.99	0.53	0.91	0.53	0.14	0.22	0.10	0.01	0.03	0.56
**EN**	0.46	0.93	0.44	0.94	0.44	0.46	0.45	0.10	0.05	0.10	0.70
**CR**	0.21	0.99	0.61	0.96	0.61	0.21	0.31	0.05	0.01	0.02	0.60

Performance metrics and variability in tuning

Macro-averaged mean absolute error (macro MAE) was used to select the optimal model using the smallest value. Values averaged across 5-fold cross-validation tests and 5 repeats.
*The final value used for the model was mtry = 42.

mtry	macro MAE	macro MSE	V3	Accuracy	Kappa	macro MAE SD	macro MSE SD	V3 SD	Accuracy SD	Kappa SD
42*	0.12954	0.07873	0.86958	0.73701	0.32762	0.00340	0.00309	0.00322	0.00469	0.01366
49	0.12968	0.07880	0.86961	0.73657	0.32843	0.00344	0.00305	0.00318	0.00515	0.01470
43	0.12978	0.07905	0.86956	0.73713	0.32809	0.00381	0.00337	0.00355	0.00527	0.01568
51	0.12983	0.07893	0.86971	0.73652	0.32967	0.00356	0.00304	0.00348	0.00556	0.01573
48	0.12986	0.07895	0.86975	0.73650	0.32818	0.00376	0.00330	0.00347	0.00533	0.01533
44	0.12987	0.07901	0.86936	0.73673	0.32745	0.00344	0.00311	0.00330	0.00493	0.01464
47	0.12990	0.07902	0.86959	0.73657	0.32779	0.00323	0.00291	0.00325	0.00506	0.01500
46	0.13000	0.07916	0.86933	0.73678	0.32816	0.00339	0.00308	0.00325	0.00506	0.01414
45	0.13007	0.07920	0.86943	0.73657	0.32701	0.00344	0.00318	0.00338	0.00498	0.01409
50	0.13008	0.07906	0.86964	0.73598	0.32802	0.00326	0.00285	0.00317	0.00507	0.01370
41	0.13012	0.07920	0.86907	0.73652	0.32517	0.00345	0.00316	0.00341	0.00503	0.01449
52	0.13017	0.07911	0.86968	0.73582	0.32784	0.00320	0.00276	0.00334	0.00524	0.01510
37	0.13019	0.07937	0.86884	0.73671	0.32436	0.00362	0.00335	0.00323	0.00524	0.01573
39	0.13022	0.07939	0.86894	0.73661	0.32458	0.00327	0.00292	0.00315	0.00497	0.01496
40	0.13022	0.07931	0.86894	0.73638	0.32435	0.00358	0.00322	0.00333	0.00516	0.01500
38	0.13051	0.07959	0.86871	0.73598	0.32265	0.00319	0.00294	0.00314	0.00496	0.01451
36	0.13074	0.07986	0.86823	0.73614	0.32207	0.00336	0.00307	0.00318	0.00494	0.01469
34	0.13076	0.07984	0.86808	0.73612	0.32068	0.00328	0.00307	0.00311	0.00477	0.01385
31	0.13086	0.07998	0.86789	0.73605	0.31883	0.00315	0.00291	0.00311	0.00473	0.01416
35	0.13090	0.08001	0.86802	0.73617	0.32069	0.00353	0.00328	0.00331	0.00489	0.01455

S2. Summary of Ordinal Forest (continued)

mtry	macro MAE	macro MSE	V3	Accuracy	Kappa	macro MAE SD	macro MSE SD	V3 SD	Accuracy SD	Kappa SD
32	0.13096	0.08003	0.86788	0.73593	0.31902	0.00303	0.00286	0.00286	0.00451	0.01283
33	0.13101	0.08008	0.86773	0.73603	0.31940	0.00345	0.00308	0.00344	0.00482	0.01415
30	0.13113	0.08029	0.86742	0.73624	0.31838	0.00317	0.00304	0.00281	0.00433	0.01273
29	0.13149	0.08052	0.86707	0.73568	0.31590	0.00326	0.00309	0.00311	0.00452	0.01368
27	0.13155	0.08065	0.86680	0.73584	0.31508	0.00323	0.00300	0.00299	0.00449	0.01354
28	0.13155	0.08063	0.86709	0.73568	0.31610	0.00352	0.00326	0.00331	0.00467	0.01434
26	0.13178	0.08086	0.86639	0.73565	0.31353	0.00337	0.00310	0.00288	0.00480	0.01439
25	0.13200	0.08105	0.86621	0.73525	0.31207	0.00344	0.00322	0.00307	0.00445	0.01333
24	0.13203	0.08113	0.86624	0.73537	0.31168	0.00290	0.00273	0.00283	0.00417	0.01299
23	0.13217	0.08132	0.86598	0.73544	0.31102	0.00321	0.00293	0.00289	0.00442	0.01321
22	0.13264	0.08165	0.86567	0.73476	0.30824	0.00309	0.00284	0.00263	0.00454	0.01344

S3. Summary of Random Forest

Confusion matrix

n test dataset

		Reference	
		**Imperiled**	**Non-imper.**
**Predicted**	**Imperiled**	359	83
	**Non-imper.**	168	1522

percentage test

		Reference	
		**Imperiled**	**Non-imper.**
**Predicted**	**Imperiled**	81.2	18.8
	**Non-imper.**	9.9	90.1

Average performance metrics and variability in tuning

Precision-recall AUC was used to select the optimal model using the largest value.

Values averaged across 5-fold cross-validation tests and 5 repeats

*The final value used for the model was mtry = 10.

mtry	V1	PR-AUC	Precision	Recall	F	ROC	Sensitivity	Specificity	Accuracy	Kappa
10*	0.72760	0.81709	0.79633	0.67013	0.72760	0.92906	0.67013	0.94353	0.87587	0.64801
9	0.72704	0.81694	0.79723	0.66852	0.72704	0.92915	0.66852	0.94400	0.87583	0.64749
11	0.72784	0.81665	0.79623	0.67061	0.72784	0.92887	0.67061	0.94347	0.87594	0.64829
12	0.72837	0.81657	0.79473	0.67259	0.72837	0.92884	0.67259	0.94275	0.87590	0.64869
13	0.72868	0.81634	0.79433	0.67335	0.72868	0.92853	0.67335	0.94257	0.87594	0.64900
8	0.72696	0.81618	0.79920	0.66701	0.72696	0.92905	0.66701	0.94481	0.87606	0.64766
7	0.72656	0.81579	0.80027	0.66559	0.72656	0.92903	0.66559	0.94528	0.87606	0.64732
14	0.72612	0.81570	0.79063	0.67165	0.72612	0.92839	0.67165	0.94142	0.87465	0.64556
16	0.72741	0.81559	0.79075	0.67373	0.72741	0.92818	0.67373	0.94129	0.87508	0.64707
6	0.72699	0.81549	0.80439	0.66351	0.72699	0.92899	0.66351	0.94683	0.87672	0.64833
15	0.72894	0.81522	0.79257	0.67506	0.72894	0.92829	0.67506	0.94182	0.87580	0.64907
17	0.72752	0.81514	0.78989	0.67458	0.72752	0.92799	0.67458	0.94089	0.87498	0.64707
20	0.72843	0.81469	0.78914	0.67667	0.72843	0.92760	0.67667	0.94045	0.87517	0.64803
19	0.72701	0.81450	0.78854	0.67468	0.72701	0.92760	0.67468	0.94039	0.87463	0.64629
18	0.72640	0.81445	0.78834	0.67373	0.72640	0.92771	0.67373	0.94042	0.87442	0.64557
5	0.72483	0.81412	0.80646	0.65859	0.72483	0.92865	0.65859	0.94789	0.87629	0.64611
4	0.72135	0.81339	0.80998	0.65064	0.72135	0.92843	0.65064	0.94967	0.87566	0.64258
3	0.71521	0.81061	0.81297	0.63881	0.71521	0.92752	0.63881	0.95156	0.87416	0.63596
2	0.70445	0.80606	0.82338	0.61599	0.70445	0.92601	0.61599	0.95642	0.87217	0.62508

S4. Variable Importance

Metadata			VIMs Random Forest					VIMs Ordinal Forest			
**Variable**	**Category**	**Subcategory**	**AUC loss**	**MDA Imperiled**	**MDA Nonimp.**	**MDBA**	**Gini**	**ER**	**RPS**	**MAE**	**MSE**
Class_count_p	Environmental	Habitat	89.4451	103.3657	75.5246	103.1459	359.2167	0.0278	0.0912	0.0893	0.2855
order	Species	Taxonomic	69.9762	88.1319	51.8204	81.7180	204.3777	0.0401	0.0942	0.1175	0.3607
Stream_pow	Environmental	Hydrology	59.9885	66.9899	52.9871	70.6240	160.9564	0.0034	0.0076	0.0088	0.0241
GDPpercent	Socioeconomic	Economy	49.7904	55.2728	44.3080	56.4182	184.1039	0.0264	0.0690	0.0825	0.2593
South_Limit	Environmental	Habitat	46.2413	40.3700	52.1126	56.7133	117.5223	0.0144	0.0355	0.0435	0.1342
Class_phys	Environmental	Habitat	45.8368	57.8966	33.7769	55.4367	103.0823	0.0122	0.0330	0.0395	0.1265
LC_PermanentWater	Environmental	Habitat	42.6819	34.3683	50.9956	57.8915	104.1624	0.0028	0.0072	0.0086	0.0270
East_Limit	Environmental	Habitat	40.8893	30.3957	51.3829	53.2616	93.0666	0.0065	0.0170	0.0192	0.0588
na_count	Socioeconomic	Knowledge	40.7704	25.6293	55.9115	59.0902	81.8569	0.0060	0.0163	0.0184	0.0581
Protected_Percent	Socioeconomic	Conservation	38.9500	36.4931	41.4069	47.8054	85.0049	0.0021	0.0052	0.0067	0.0211
hfp_2009	Socioeconomic	Footprint	38.9373	35.0967	42.7780	51.3233	72.1162	0.0190	0.0451	0.0531	0.1559
bio_8	Environmental	Climate	37.2478	30.5617	43.9340	49.7565	73.3646	0.0028	0.0065	0.0080	0.0245
Log_Q_avg	Environmental	Hydrology	35.1145	24.7696	45.4595	52.5142	68.3499	0.0014	0.0038	0.0040	0.0129
bio_18	Environmental	Climate	34.9761	29.7402	40.2120	51.0194	62.1359	0.0015	0.0038	0.0043	0.0130
EarlyPC	Socioeconomic	Economy	34.7879	38.6830	30.8927	40.2628	62.1903	0.0116	0.0272	0.0304	0.0850
bio_19	Environmental	Climate	34.6768	30.2012	39.1525	47.6496	68.1379	0.0031	0.0077	0.0093	0.0290
LC_SeasonalWater	Environmental	Habitat	34.4684	27.5468	41.3901	47.8605	68.3710	0.0011	0.0026	0.0032	0.0098
bio_16	Environmental	Climate	33.1319	25.0688	41.1950	47.8033	64.6145	0.0024	0.0056	0.0068	0.0203
bio_4	Environmental	Climate	32.2146	23.9822	40.4470	48.2138	65.5232	0.0026	0.0064	0.0079	0.0236
LC_Tree	Environmental	Habitat	31.8208	25.8667	37.7749	45.2584	60.2192	0.0021	0.0055	0.0066	0.0205
LC_Shrub	Environmental	Habitat	31.7301	20.7433	42.7169	46.8885	63.0227	0.0038	0.0095	0.0115	0.0348
CMI_indx	Environmental	Climate	31.7300	26.1796	37.2805	45.2262	57.2702	0.0008	0.0020	0.0024	0.0074
Log_Q_var	Environmental	Hydrology	31.5680	20.3580	42.7781	50.5392	58.6378	0.0009	0.0024	0.0028	0.0082
Ramsar_count_p	Socioeconomic	Conservation	30.8820	23.8354	37.9285	35.6146	97.4943	0.0012	0.0034	0.0036	0.0099
bio_17	Environmental	Climate	30.3738	24.1812	36.5663	45.9372	54.7505	0.0009	0.0021	0.0025	0.0071
GDPslope	Socioeconomic	Economy	29.8744	27.1654	32.5835	38.5484	45.9212	0.0030	0.0068	0.0080	0.0224
PDslope	Socioeconomic	Development	28.7051	17.8375	39.5727	45.0044	58.9026	0.0016	0.0041	0.0045	0.0135
Reach_type	Environmental	Hydrology	27.4818	33.2123	21.7512	32.4255	45.8992	0.0018	0.0057	0.0063	0.0207

S4. Variable Importance (continued)

Metadata			VIMs Random Forest					VIMs Ordinal Forest			
Variable	Category	Subcategory	AUC loss	MDA Imperiled	MDA Nonimperil.	MDBA	Gini	ER	RPS	MAE	MSE
LC_Grass	Environmental	Habitat	27.2377	12.6401	41.8353	45.9886	57.3187	0.0029	0.0068	0.0088	0.0277
PDpercent	Socioeconomic	Development	26.3006	15.0308	37.5703	41.1997	55.7420	0.0007	0.0020	0.0021	0.0060
Mean_Elevation	Environmental	Habitat	26.2031	17.2932	35.1129	36.6713	54.6780	0.0009	0.0022	0.0026	0.0086
LC_Crops	Socioeconomic	Development	25.5835	16.6356	34.5315	38.0934	62.3754	0.0024	0.0056	0.0064	0.0185
POP_SERVED_p	Socioeconomic	Footprint	23.9127	22.0798	25.7457	27.3558	51.3634	0.0006	0.0017	0.0020	0.0065
CAB_Dams_p	Socioeconomic	Impoundments	19.9681	21.1146	18.8216	21.9484	44.3962	0.0013	0.0033	0.0038	0.0119
Dams_p	Socioeconomic	Impoundments	19.8966	16.9914	22.8018	24.6696	40.3295	0.0006	0.0017	0.0017	0.0050
RA_Dams_p	Socioeconomic	Impoundments	17.5345	16.4926	18.5763	20.8521	39.3541	0.0076	0.0168	0.0197	0.0544
Extir_count	Environmental	Biotic	16.8406	11.2588	22.4225	24.7152	14.7531	0.0044	0.0111	0.0135	0.0409
LC_Snow	Environmental	Hydrology	13.7559	10.7756	16.7361	20.3232	8.9912	0.0004	0.0011	0.0013	0.0041
Class_hydr	Environmental	Habitat	12.9454	9.9644	15.9264	19.5867	9.7523	0.0012	0.0032	0.0034	0.0104
EnvTemp	Environmental	Habitat	12.8897	11.5170	14.2623	18.8528	11.5310	0.0004	0.0010	0.0012	0.0032
Brack	Species	Physiology	12.6191	13.4212	11.8169	16.5933	5.4429	0.0016	0.0050	0.0057	0.0191
LengthCategory	Species	Life history	11.4834	14.2664	8.7003	16.6568	14.2346	0.0003	0.0011	0.0010	0.0032
DemersPelag	Species	Physiology	11.3432	8.1399	14.5465	16.1843	16.7485	0.0008	0.0019	0.0022	0.0062
Electrogenic	Species	Life history	11.0653	20.3778	1.7527	17.9089	4.5174	0.0004	0.0013	0.0017	0.0068
GameFish	Socioeconomic	Value	10.5103	15.1735	5.8471	11.8343	2.8810	0.0001	0.0002	0.0001	0.0003
BodyShapeI	Species	Life history	8.9995	9.6074	8.3916	13.2664	17.4837	0.0005	0.0014	0.0014	0.0041
Saltwater	Species	Physiology	7.5320	10.1471	4.9170	9.6015	1.8923	0.0003	0.0009	0.0009	0.0029
Aquarium	Socioeconomic	Value	6.9155	6.1338	7.6971	10.3771	7.1535	0.0002	0.0005	0.0006	0.0019
UsedforAquaculture	Socioeconomic	Value	4.5985	5.2660	3.9309	5.6962	2.9320	0.0000	0.0002	0.0000	0.0000
Dangerous	Species	Life history	1.7009	3.2421	0.1597	1.4096	0.9300	0.0000	0.0000	0.0000	0.0000
UsedasBait	Socioeconomic	Value	1.3984	0.1984	2.5983	2.0302	0.2949	0.0000	0.0000	0.0000	0.0000
Class_geom	Environmental	Habitat	0.4243	0.5716	0.2770	0.5066	1.4082	0.0000	0.0000	0.0000	0.0000

Table S5. Partial Dependence on Binary Classification Model

Variable importance		Metadata					Partial Dependence on binary classification model
**Rank**	**AUC loss**	**Variable**	**Category**	**Subcategory**	**Meaning**	**Unit**	
1	89.45	Class_count_p	Environmental	Habitat	Count of different classes of geomorphic sub-classification / range area	count / km2	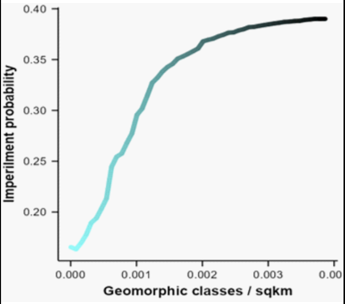
2	69.98	Order	Species	Taxonomy	Taxonomic order	categorical	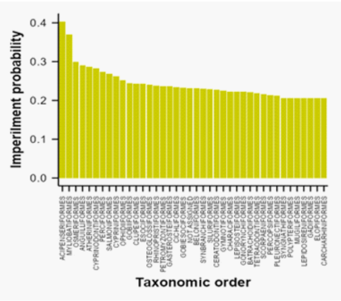
3	59.99	Stream_pow	Environmental	Hydrology	Total stream power	kW/m2	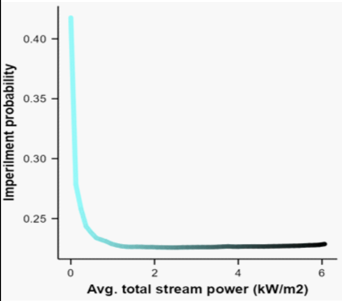
4	49.79	GDPpercent	Social	Economy	Avg. annual percent change in GDP	percent	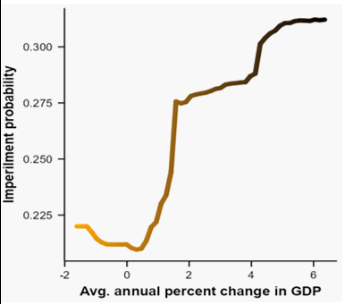
5	45.84	Class_phys	Environmental	Habitat	Mode of classes of physio-climatic sub-classification	categorical	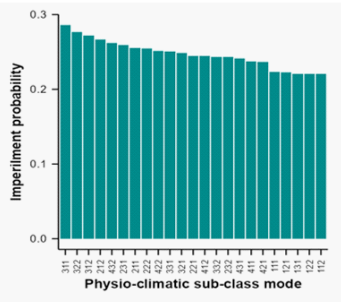

Table S5. Partial Dependence on Binary Classification Model (continued)

Variable importance		Metadata					Partial Dependence on binary classification model
AUC loss	Rank*	Variable	Category	Subcategory	Meaning	Unit	
6	42.68	LC_PermanentWater	Environmental	Habitat	Cover type percentage	percent	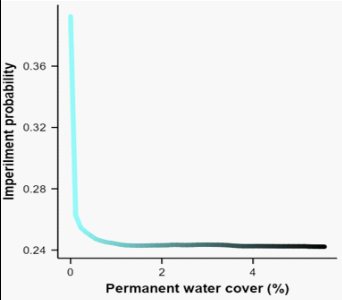
7	40.89	East_Limit	Environmental	Habitat	Species range eastern limit	decimal degrees	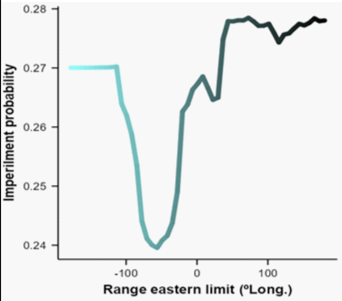
8	40.77	na_count	Social	Knowledge	Knowledge gaps	count	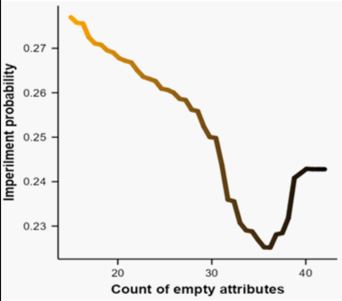
9	38.95	Protected_Percent	Social	Conservation	percent of area protected within species range	percent	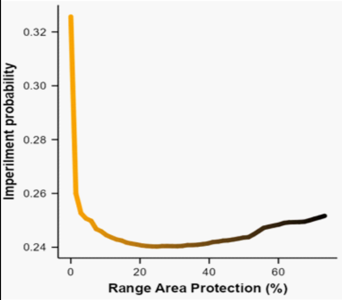
10	38.94	hfp_2009	Social	Footprint	Human-footprint index in 2009	Index	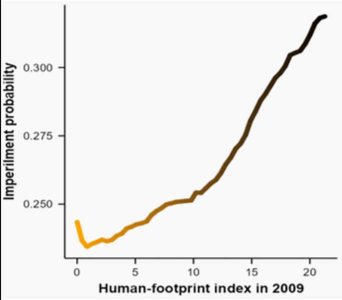

Table S5. Partial Dependence on Binary Classification Model (continued)

Variable importance		Metadata					Partial Dependence on binary classification model
AUC loss	Rank*	Variable	Category	Subcategory	Meaning	Unit	
11	37.25	bio_8	Environmental	Climate	Historic Mean Temperature of Wettest Quarter	°C	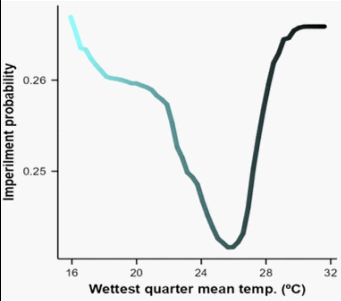
12	35.11	Log_Q_avg	Environmental	Hydrology	log10 of long-term average discharge	m3/sec	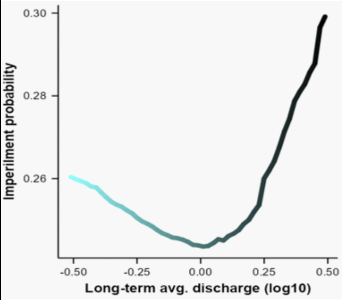
13	34.98	bio_18	Environmental	Climate	Historic Precipitation of Warmest Quarter	mm	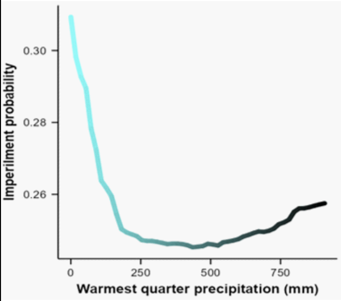
14	34.79	EarlyPC	Social	Economy	Early per capita GDP (1960s)	current USD (millions)	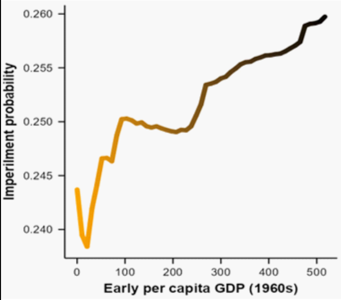
15	34.68	bio_19	Environmental	Climate	Historic Precipitation of Coldest Quarter	mm	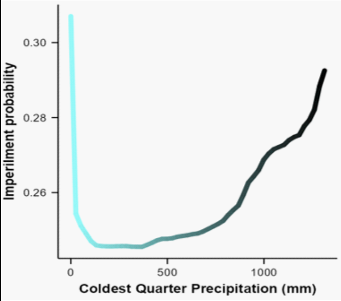

Table S5. Partial Dependence on Binary Classification Model (continued)

Variable importance		Metadata					Partial Dependence on binary classification model
AUC loss	Rank*	Variable	Category	Subcategory	Meaning	Unit	
16	34.47	LC_SeasonalWater	Environmental	Habitat	Cover type percentage	Percent	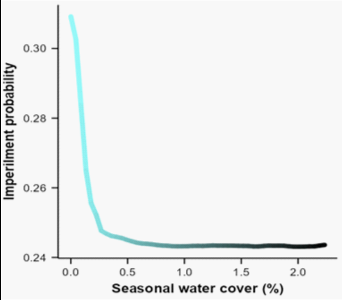
17	33.13	bio_16	Environmental	Climate	Historic Precipitation of Wettest Quarter	mm	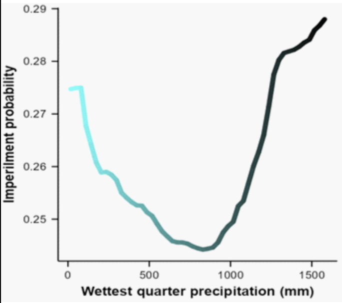
18	32.21	bio_4	Environmental	Climate	Historic Temperature Seasonality	°C (SD)	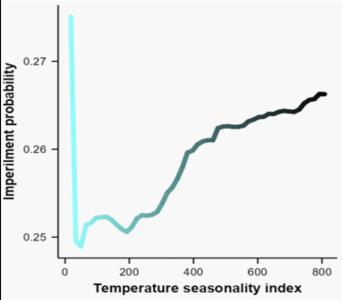
19	31.82	LC_Tree	Environmental	Habitat	Cover type percentage	Percent	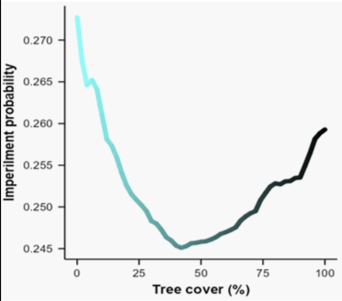
20	31.73	LC_Shrub	Environmental	Habitat	Cover type percentage	Percent	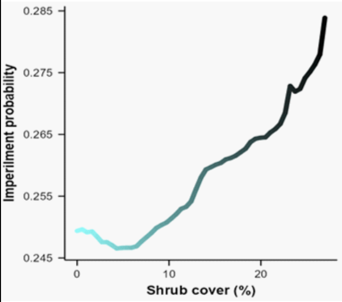

Table S5. Partial Dependence on Binary Classification Model (continued)

Variable importance		Metadata					Partial Dependence on binary classification model
AUC loss	Rank*	Variable	Category	Subcategory	Meaning	Unit	
21	31.73	CMI_indx	Environmental	Climate	Mean reach climate moisture index	index	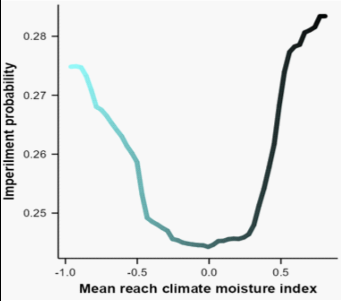
22	31.57	Log_Q_var	Environmental	Hydrology	Flow regime variability as log10 of maximum monthly avg. / long term avg. discharge	index	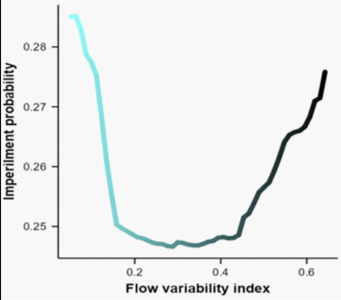
23	30.88	Ramsar_count_p	Social	Conservation	number of Ramsar sites / range area	count / area	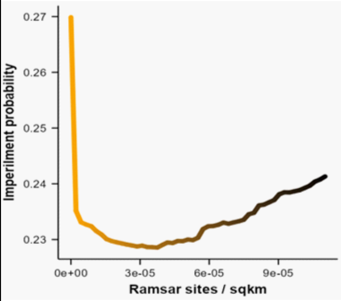
24	30.37	bio_17	Environmental	Climate	Historic Precipitation of Driest Quarter	mm	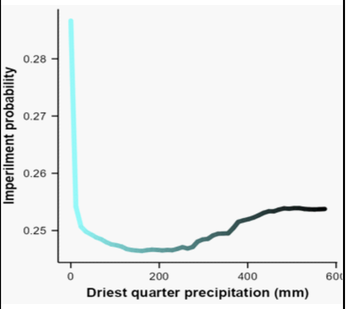
25	29.87	GDPslope	Social	Economy	Avg. annual change in GDP	current USD (millions)	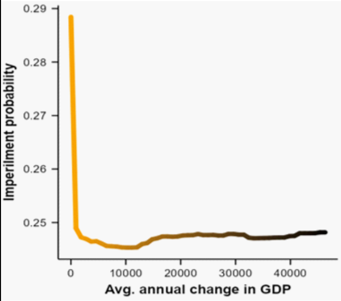

Table S5. Partial Dependence on Binary Classification Model (continued)

**Variable importance**		**Metadata**					**Partial Dependence on binary classification model**
**AUC loss**	**Rank***	**Variable**	**Category**	**Subcategory**	**Meaning**	**Unit**	
26	28.71	PDslope	Social	Development	Avg. annual change in population density 2000-2020	population/km2	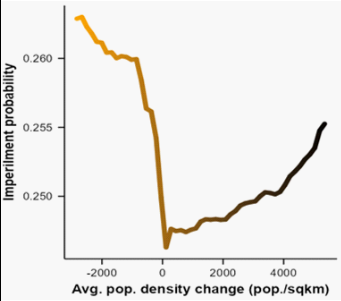
27	27.48	Reach_type	Environmental	Hydrology	Mode of reach type (4 classes)	categorical	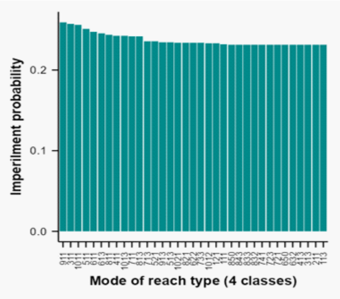
28	27.24	LC_Grass	Environmental	Habitat	Cover type percentage	percent	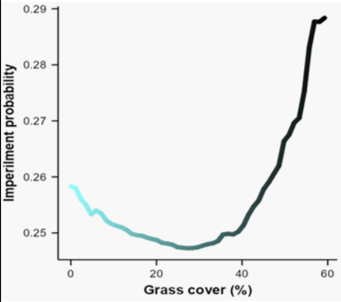
29	26.30	PDpercent	Social	Development	Avg. annual percent change in population density	percent	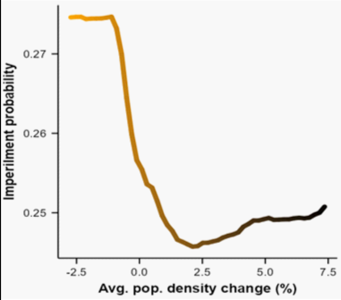
30	25.58	LC_Crops	Social	Development	Cover type percentage	Percent	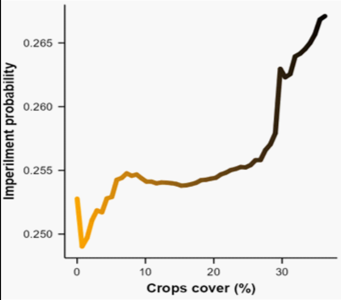

Table S5. Partial Dependence on Binary Classification Model (continued)

Variable importance		Metadata					Partial Dependence on binary classification model
**AUC loss**	**Rank***	**Variable**	**Category**	**Subcategory**	**Meaning**	**Unit**	
31	23.91	POP_SERVED_p	Social	Footprint	human population wastewater service/area of species range	Population	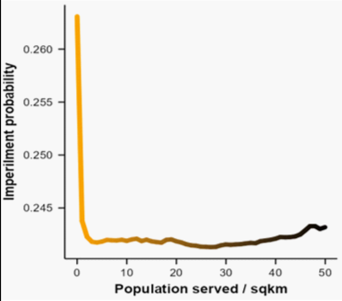
32	19.97	CAB_Dams_p	Social	Impoundments	Total catchment areas blocked / area of species range	Proportion	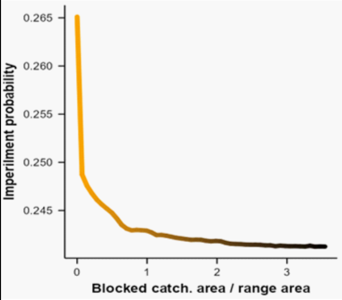
33	19.90	Dams_p	Social	Impoundments	Total existing large dams within range / range area	Count/km2	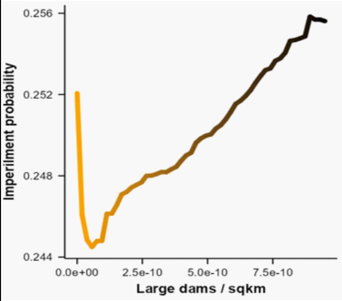
34	17.53	RA_Dams_p	Social	Impoundments	Total reservoir area in range / area of species range	Proportion	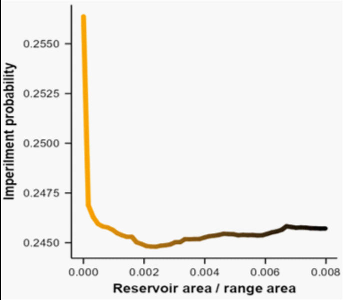
35	16.84	Extir_count	Environmental	Biotic	count of extirpated species in range	count	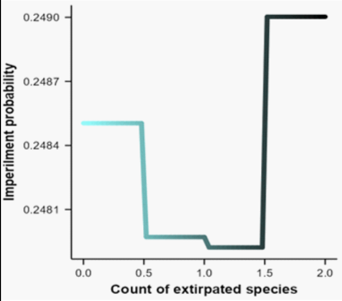

Table S5. Partial Dependence on Binary Classification Model (continued)

Variable importance		Metadata					Partial Dependence on binary classification model
AUC loss	Rank*	Variable	Category	Subcategory	Meaning	Unit	
36	13.76	LC_Snow	Environmental	Hydrology	Cover type percentage	percent	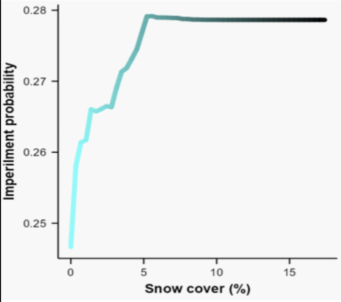
37	12.95	Class_hydr	Environmental	Habitat	Mode of classes of hydrologic sub-classification	categorical	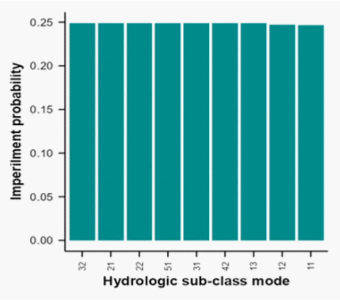
38	12.89	EnvTemp	Environmental	Habitat	Tropical, subtropical, temperate	categorical	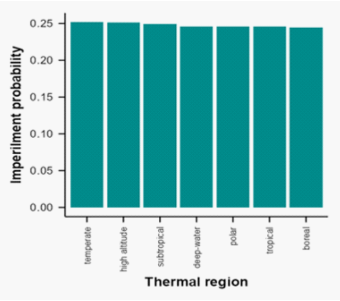
39	12.62	Brack	Species	Physiology	Brackishwater	presence	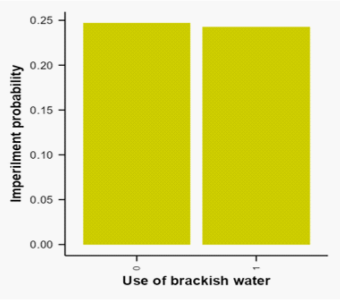
40	11.48	LengthCategory	Species	Life history	Small, medium, large	categorical	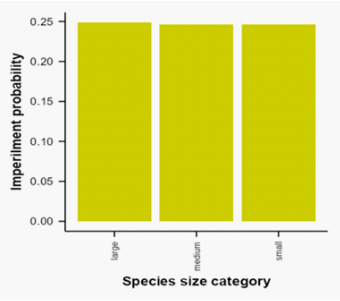

Table S5. Partial Dependence on Binary Classification Model (continued)

Variable importance		Metadata					Partial Dependence on binary classification model
AUC loss	Rank*	Variable	Category	Subcategory	Meaning	Unit	
41	11.34	DemersPelag	Species	Physiology	Demersal/Pelagic	categorical	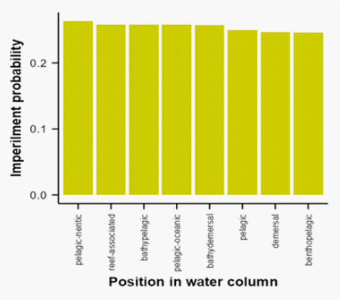
42	11.07	Electrogenic	Species	Life history	Electric	categorical	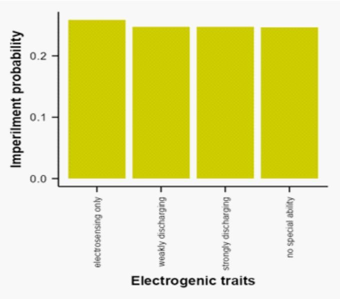
43	10.51	GameFish	Social	Value	Game fish	presence	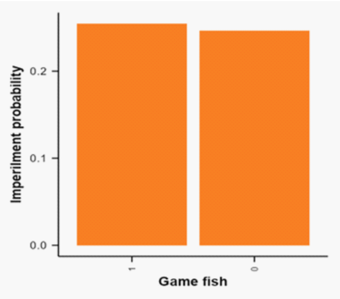
44	9.00	BodyShapeI	Species	Life history	Body form	categorical	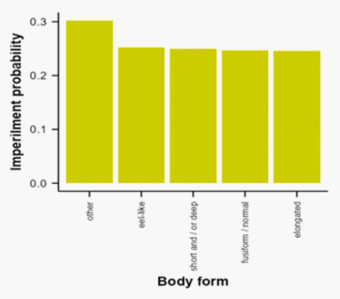
45	7.53	Saltwater	Species	Physiology	Saltwater	presence	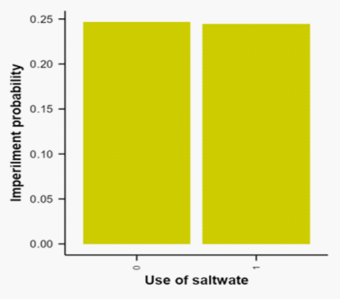

Table S5. Partial Dependence on Binary Classification Model (continued)

Variable importance		Metadata					Partial Dependence on binary classification model
AUC loss	Rank*	Variable	Category	Subcategory	Meaning	Unit	
46	6.92	Aquarium	Social	Value	Aquarium trade	categorical	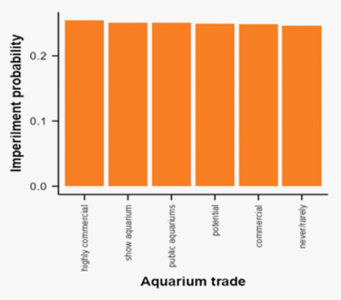
47	4.60	UsedforAquaculture	Social	Value	Aquaculture	categorical	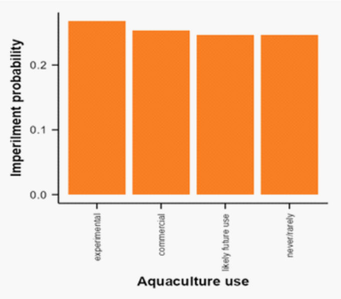
48	1.70	Dangerous	Species	Life history	Dangerous	categorical	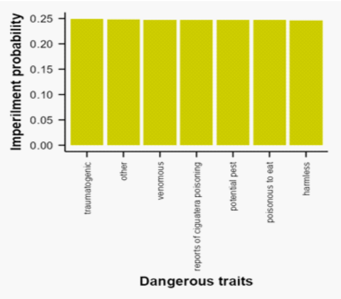
49	1.40	UsedasBait	Social	Value	Used as bait	categorical	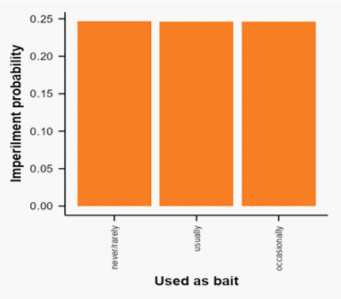
50	0.42	Class_geom	Environmental	Habitat	Mode of different classes of geomorphic sub-classification	categorical	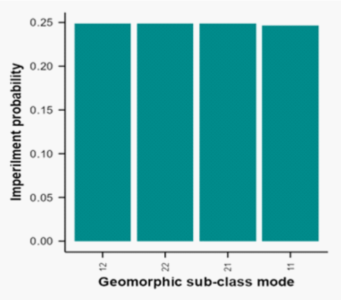
