# Supplementary material for: Environment, taxonomy, and socioeconomics predict non-imperilment in freshwater fishes
Source: Nat Commun. 2026 Feb 16;17:1661. doi: 10.1038/s41467-025-68154-w (PMC12909873; doi:10.1038/s41467-025-68154-w)
Supplement: Supplementary file 2 — Reporting Summary [file 41467_2025_68154_MOESM2_ESM.pdf]

nature portfolio

Corresponding author(s): Christina Murphy

Last updated by author(s): Aug 27, 2025

Reporting Summary

Nature Portfolio wishes to improve the reproducibility of the work that we publish. This form provides structure for consistency and transparency in reporting. For further information on Nature Portfolio policies, see our Editorial Policies and the Editorial Policy Checklist.

Statistics

For all statistical analyses, confirm that the following items are present in the figure legend, table legend, main text, or Methods section.

n/a	Confirmed	
x	☐	The exact sample size (*n*) for each experimental group/condition, given as a discrete number and unit of measurement
x	☐	A statement on whether measurements were taken from distinct samples or whether the same sample was measured repeatedly
x	☐	The statistical test(s) used AND whether they are one- or two-sided<br>*Only common tests should be described solely by name; describe more complex techniques in the Methods section.*
x	☐	A description of all covariates tested
x	☐	A description of any assumptions or corrections, such as tests of normality and adjustment for multiple comparisons
x	☐	A full description of the statistical parameters including central tendency (e.g. means) or other basic estimates (e.g. regression coefficient) AND variation (e.g. standard deviation) or associated estimates of uncertainty (e.g. confidence intervals)
x	☐	For null hypothesis testing, the test statistic (e.g. *F*, *t*, *r*) with confidence intervals, effect sizes, degrees of freedom and *P* value noted<br>*Give P values as exact values whenever suitable.*
x	☐	For Bayesian analysis, information on the choice of priors and Markov chain Monte Carlo settings
☐	x	For hierarchical and complex designs, identification of the appropriate level for tests and full reporting of outcomes
☐	x	Estimates of effect sizes (e.g. Cohen's *d*, Pearson's *r*), indicating how they were calculated

Our web collection on statistics for biologists contains articles on many of the points above.

Software and code

Policy information about availability of computer code

Data collection	WThe data used in this study were sourced from publicly available datasets as described in Table 1 (main text) and Methods. Code for compiling data are here: https://github.com/AndresOlivos/freshwater_fish_imperilment_classification and archived in https://doi.org/10.5281/zenodo.17674411.
Data analysis	Two software were used for data management and analysis: ArcGIS Pro and R as detailed in the methods. All code for compiling datasets, running analyses, and result tables can be found here: https://github.com/AndresOlivos/freshwater_fish_imperilment_classification and archived in https://doi.org/10.5281/zenodo.17674411.

For manuscripts utilizing custom algorithms or software that are central to the research but not yet described in published literature, software must be made available to editors and reviewers. We strongly encourage code deposition in a community repository (e.g. GitHub). See the Nature Portfolio guidelines for submitting code & software for further information.

Data

Policy information about availability of data

All manuscripts must include a data availability statement. This statement should provide the following information, where applicable:

- Accession codes, unique identifiers, or web links for publicly available datasets
- A description of any restrictions on data availability
- For clinical datasets or third party data, please ensure that the statement adheres to our policy

Data are from publicly available databases: see Methods for sources. Scripts and tables can be accessed at https://github.com/AndresOlivos/

freshwater_fish_imperilment_classification and archived in https://doi.org/10.5281/zenodo.17674411. Data questions can be addressed to J. Andres Olivos (andres.olivos@oregonstate.edu).

Research involving human participants, their data, or biological material

Policy information about studies with human participants or human data. See also policy information about sex, gender (identity/presentation), and sexual orientation and race, ethnicity and racism.

Reporting on sex and gender	Study did not include human participants, their data, or biological material.
Reporting on race, ethnicity, or other socially relevant groupings	Study did not include human participants, their data, or biological material.
Population characteristics	Study did not include human participants, their data, or biological material.
Recruitment	Study did not include human participants, their data, or biological material.
Ethics oversight	Study did not include human participants, their data, or biological material.

Note that full information on the approval of the study protocol must also be provided in the manuscript.

Field-specific reporting

Please select the one below that is the best fit for your research. If you are not sure, read the appropriate sections before making your selection.

☐ Life sciences ☐ Behavioural & social sciences ☒ Ecological, evolutionary & environmental sciences

For a reference copy of the document with all sections, see nature.com/documents/nr-reporting-summary-flat.pdf

Ecological, evolutionary & environmental sciences study design

All studies must disclose on these points even when the disclosure is negative.

Study description	Here, we developed a machine-learning model to predict the conservation status of freshwater fishes globally using a comprehensive database encompassing extrinsic environmental and socioeconomic factors and intrinsic species-specific characteristics.
Research sample	The model was able to classify 10,631 freshwater fish species using 52 variables from 12 global sources.
Sampling strategy	We aggregated and coded imperilment as 1 and 0, representing imperiled and non-imperiled, respectively, and constructed a binary random forest model using the 'randomForest' package in R with the unit of analysis being species status. We also present results from ordinal forest modeling.
Data collection	We used all freshwater fishes listed by IUCN RedList 2024 (category as response variable) and 52 explanatory variables queried from 12 global sources (Table 1 and Table S1).
Timing and spatial scale	IUCN RedList categories for freshwater fishes from 2024 were used as response variable globally. Explanatory variables varied in their timing and scales, with the most recent period available being used at the time of modeling (2025).
Data exclusions	Species classified by IUCN RedList as Data Deficient or Extinct.
Reproducibility	All settings are detailed in themethods and full code is available here: https://gitfront.io/r/AndresOlivos/BTK8dxjNenih/freshwater-fish-imperilment-classification/
Randomization	We constructed a binary random forest model calling the randomForest package through caret for repeated cross validation and hyperparameter tuning. We also used training and test datasets. For the training dataset, each tree used 63.2% of the data with the remaining 36.8% of the data used to assess predictive performance.
Blinding	Blinding was not relevant for this study.

Did the study involve field work? ☐ Yes ☒ No

Reporting for specific materials, systems and methods

We require information from authors about some types of materials, experimental systems and methods used in many studies. Here, indicate whether each material, system or method listed is relevant to your study. If you are not sure if a list item applies to your research, read the appropriate section before selecting a response.

Materials & experimental systems

n/a	Involved in the study
☒	☐ Antibodies
☒	☐ Eukaryotic cell lines
☒	☐ Palaeontology and archaeology
☒	☐ Animals and other organisms
☒	☐ Clinical data
☒	☐ Dual use research of concern
☒	☐ Plants

Methods

n/a	Involved in the study
☒	☐ ChIP-seq
☒	☐ Flow cytometry
☒	☐ MRI-based neuroimaging

Plants

Seed stocks	Study did not include plants.
Novel plant genotypes	Study did not include plants.
Authentication	Study did not include plants.
